# Antimicrobial and antibiofilm activities of formylchromones against *Vibrio parahaemolyticus* and *Vibrio harveyi*

**DOI:** 10.3389/fcimb.2023.1234668

**Published:** 2023-08-17

**Authors:** Ezhaveni Sathiyamoorthi, Jin-Hyung Lee, Yulong Tan, Jintae Lee

**Affiliations:** ^1^ School of Chemical Engineering, Yeungnam University, Gyeongsan, Republic of Korea; ^2^ Special Food Research Institute, Qingdao Agricultural University, Qingdao, China

**Keywords:** chromones, antibacterial, biofilm, *V. harveyi*, *Vibrio parahaemolyticus*, virulence factors

## Abstract

Gram-negative *Vibrio* species are major foodborne pathogens often associated with seafood intake that causes gastroenteritis. On food surfaces, biofilm formation by *Vibrio* species enhances the resistance of bacteria to disinfectants and antimicrobial agents. Hence, an efficient antibacterial and antibiofilm approach is urgently required. This study examined the antibacterial and antivirulence effects of chromones and their 26 derivatives against *V. parahaemolyticus* and *V. harveyi*. 6-Bromo-3-formylchromone (6B3FC) and 6-chloro-3-formylchromone (6C3FC) were active antibacterial and antibiofilm compounds. Both 6B3FC and 6C3FC exhibited minimum inhibitory concentrations (MICs) of 20 µg/mL for planktonic cell growth and dose-dependently inhibited biofilm formation. Additionally, they decreased swimming motility, protease activity, fimbrial agglutination, hydrophobicity, and indole production at 20 µg/mL which impaired the growth of the bacteria. Furthermore, the active compounds could completely inhibit the slimy substances and microbial cells on the surface of the squid and shrimp. The most active compound 6B3FC inhibited the gene expression associated in quorum sensing and biofilm formation (*luxS*, *opaR*), pathogenicity (*tdh*), and membrane integrity (*vmrA*) in *V. parahaemolyticus*. However, toxicity profiling using seed germination and *Caenorhabditis elegans* models suggests that 6C3FC may have moderate effect at 50 µg/mL while 6B3FC was toxic to the nematodes 20-100 µg/mL. These findings suggest chromone analogs, particularly two halogenated formylchromones (6B3FC and 6C3FC), were effective antimicrobial and antibiofilm agents against *V. parahaemolyticus* in the food and pharmaceutical sectors.

## 1 Introduction

Foodborne pathogens have been a major concern in raw aquatic products. Vibriosis is a bacterial illness that significantly impacts mariculture globally, causing significant mortality rates. In seawater, *Vibrio anguillarum* (Zammuto et al., 2022), *Vibrio parahaemolyticus*, and *Vibrio vulnificus* (Nithya and Pandian, 2010) are the most common pathogens. In contrast, *Vibrio mimicus* (Li et al., 2020) and *Vibrio cholerae* (Silva and Benitez, 2016) are most common in freshwater. Among them, *V. parahaemolyticus*, a Gram-negative bacterium, is found in various marine and freshwater products, including shrimp, crabs, and oysters (Mahmoud, 2014; Liu et al., 2022). More gravely, it is the prominent cause of seafood-related contagions and mortality globally, encompassing three categories of diseases: septicemia, infections in wound, and gastroenteritis (Kim et al., 2022a). *V. parahaemolyticus* can produce thermostable direct hemolysin (TDH) that can lyse red blood cells, which are frequently linked to strains recovered from people with gastroenteritis. As a result, TDH has been identified as the primary virulence factor of *V. parahaemolyticus* (Joseph et al., 1982). Other species-specific genetic markers for identifying *V. parahaemolyticus* have been found in addition to the *trh, tlh*, and *tdh* genes, the virulence factors of *V. parahaemolyticus* (Bej et al., 1999).

Chromones and their derivatives are heterocyclic in structure and phenolic compounds that contain oxygen as a heteroatom and have a benzo-pyrone structure. Chromone is a naturally occurring chemical found in both human and animal diets that is less harmful to mammalian cells (Mohsin et al., 2020). Chromones are abundant in many plant genera, including Aquilaria, Aloe, Hypericum, Polygonum, and Cassia (Semwal et al., 2020). Chromone compounds have antibacterial (Sharma and Kumar, 2014), antifungal (Hussain et al., 2007), antioxidant, and antimicrobial activities (Asamenew et al., 2011), with potential in medicinal and pharmacology. Different substituents have been attached to the benzene or pyrone rings to modify the chromone scaffold as a pharmacophore of many natural and manufactured bioactive compounds. It is also the fundamental nucleus of flavones, which are well-known for their antioxidant properties.

This study tested 26 chromone derivatives and chromone for their ability to combat two *V. parahaemolyticus* and *V. harveyi*. Further research was conducted on the impact of the most active compounds, 6-bromo-3-formyl chromone (6B3FC) and 6-chloro-3-formyl chromone (6C3FC), on biofilm formation and virulence factors. Scanning electron microscopy (SEM) and live cell microscopy were used to examine the phenotypic switching and cell morphology of *V. parahaemolyticus* on the surface of squid and shrimp. Motility, aggregation, protease, and indole assays were performed to establish the inhibitory effects on *V. parahaemolyticus*. The molecular basis of their activity was studied using a quantitative real-time polymerase chain reaction (qRT-PCR). In addition, their toxicity and ecological effects were investigated using nematode models and seed germination. The result of the study will improve seafood biofilm decontamination and reduce the risk of pathogenic infections. It will aid in overcoming the limitations of the existing chemical disinfection treatments and establishing novel cost effective control systems for the safety and quality of marine food products.

## 2 Materials and methods

### 2.1 Strains and chemicals

The bacterial strains used were *V. parahaemolyticus* strain ATCC 17802 and *V. harveyi* ATCC 14126, both obtained from the American collection culture center (Manassas, USA). The bacteria were cultured in Luria–Bertani media with 3% NaCl (w/v), named as marine Luria–Bertani (mLB). All the experiments were carried out at 30°C in mLB liquid media and solid agar plates. Chromones (CHR) and its twenty-six derivatives were acquired from Sigma–Aldrich (St. Louis, USA), or Combi-Blocks, Inc. (San Diego, USA) (**Table 1**): 2-amino-3-formylchromone, 2-amino-6-chloro-3-formylchromone, 3-bromochromone, 6-bromo-4H-chromen-4-one, 6-bromochromone-2-carboxylic acid, 6-bromochromone-3-carbonitrile, 6-bromo-3-formylchromone, 3-bromo-6-chlorochromone, chromone-2-carboxylic acid, chromone-3-carboxylic acid, chromone-3-carbonitrile, 6-chloro-3-formylchromone, 6-chlorochromone, 6-chlorochromone-2-carboxylic acid, 6-chloro-7-methylchromone, 6,8-dichlorochromone-3-carbonitrile, 3-formyl-6-isopropylchromone, 3-formyl-6-nitrochromone, 3-formyl-6-methylchromone, 6-formyl-6-methoxychromone, 6-fluorochromone-2-carboxylic acid, 6-isopropylchromone-3-carbonitrile, 6-methylchromone, 6-methylchromone-3-carbonitrile, 6-methylchromone-2-carboxylic acid, and 6-nitrochromone. Dimethyl sulfoxide (DMSO) was used to dissolve these chromones and their derivatives. DMSO at a 0.1% v/v concentration used as a negative control. Freshly frozen squid (*Todarodes pacificus*) were procured from domestic market (Gyeongsan, South Korea), and transferred to the testing facilities under refrigeration, and stored at a temperature of −20°C for the purpose of conducting a biotic surface test.

**Table 1:** Minimum inhibitory concentration (MICs) of chromone and its derivatives against *V. parahaemolyticus* and *V. harveyi*.

No	Compounds	Structure	*V. parahaemolyticus*			*V. harveyi*		
			MIC (µg/mL)	Biofilm (%)		MIC (µg/mL)	Biofilm(%)	
				20	50		20	50
1.	2-Amino-3-formylchromone	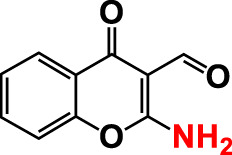	>500	126.9	124.6	>500	115.9	103.2
2.	2-Amino-6-chloro-3-formylchromone	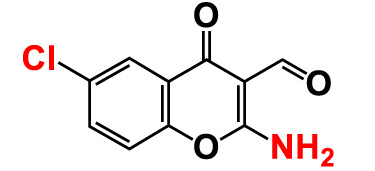	>500	128.7	123.8	>500	114.3	112.2
3.	3-Bromochromone	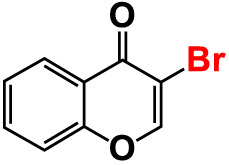	200	129.4	116.5	200	114.8	99.0
4.	6-Bromo-4H-chromen-4-one	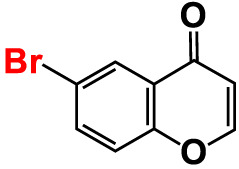	>500	126.3	95.5	>500	113.1	100.1
5.	6-Bromochromone-2-carboxylic acid	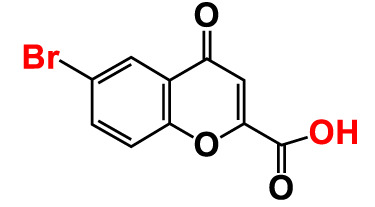	>500	127.9	128.4	>500	115.7	116.0
6.	6-Bromochromone-3-carbonitrile	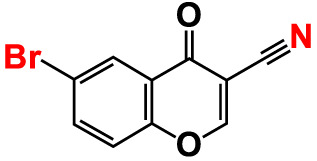	>500	129.5	127.1	>500	115.4	115.2
**7. **	**6-Bromo-3-formylchromone (6B3FC)**	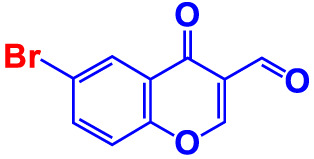	**20**	**8.8**	**3.24**	**20**	**8.9**	**1.41**
8.	3-Bromo-6-chlorochromone	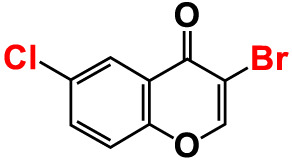	75	118.8	11.9	50	83.0	112.1
9.	**Chromone**	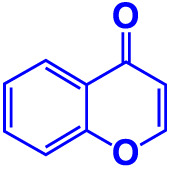	**>500**	**129.0**	**129.5**	**>500**	**114.1**	**113.7**
10.	Chromone-2-carboxylic acid	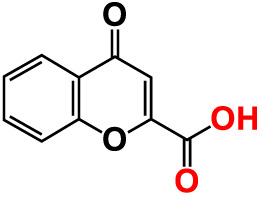	>500	129.0	129.4	>500	114.4	115.6
11.	Chromone-3-carboxylic acid	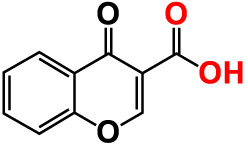	>500	129.2	122.3	>500	115.7	111.3
12.	Chromone-3-carbonitrile	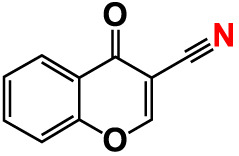	400	127.9	129.0	400	114.0	103.4
**13. **	**6-Chloro-3-formylchromone (6C3FC)**	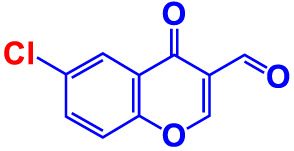	**20**	**1.2**	**1.8**	**20**	**0.4**	**0.18**
14.	6-Chlorochromone	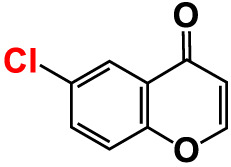	>500	129.4	121.1	>500	116.1	100.6
15.	6-Chlorochromone-2-carboxylic acid	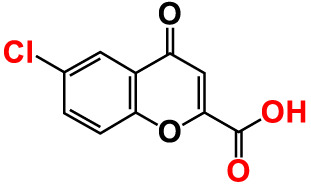	>500	127.3	128.2	>500	116.0	114.6
16.	6-Chloro-7-methylchromone	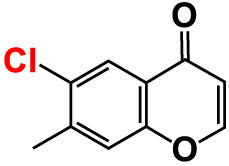	>500	126.5	71.1	>500	115.8	75.1
17.	6,8-Dichlorochromone-3-carbonitrile	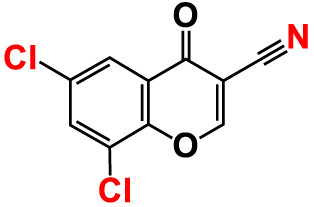	>500	118.5	84.3	>500	110.8	94.0
18.	3-Formyl-6-isopropylchromone	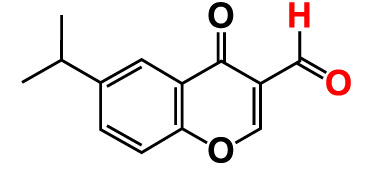	>500	34.9	2.4	>500	41.8	1.2
19.	3-Formyl-6-nitrochromone	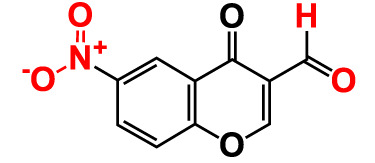	50	124.1	97.0	20	116.8	94.6
20.	3-Formyl-6-methylchromone	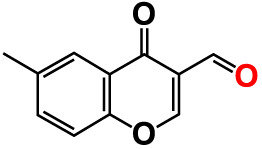	>500	39.4	1.0	>500	55.7	2.4
21.	3-Formyl-6-methoxychromone	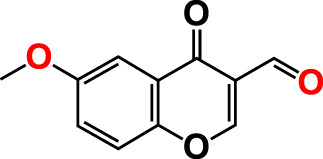	50	109.0	46.3	50	104.5	65.1
22.	6-Fluorochromone-2-carboxylic acid	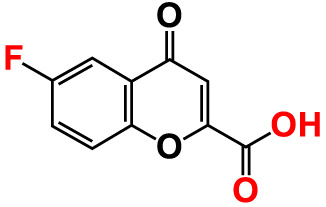	75	128.7	126.8	75	116.7	115.1
23.	6-Isopropylchromone-3-carbonitrile	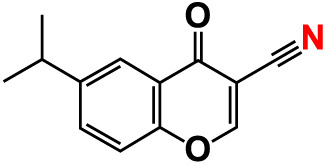	>500	110.8	54.4	>500	97.0	52.9
24.	6-Methylchromone	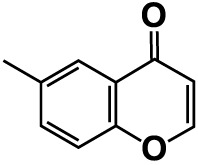	50	126.7	123.0	200	116.3	115.3
25.	6-Methylchromone-3-carbonitrile	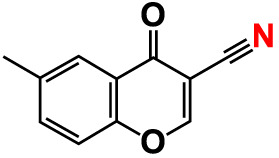	75	129.2	127.7	75	116.1	116.5
26.	6-Methylchromone-2-carboxylic acid	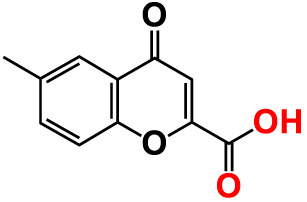	>500	127.9	128.3	>500	116.2	115.1
27.	6-NitroChromone	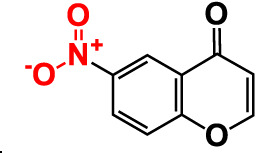	200	127.2	121.9	200	115.3	112.2

The chromone and two most active hits were presented in blue letter.

### 2.2 The growth of bacterial cells and minimum inhibitory concentration (MIC)

The grown overnight bacterial culture was reinoculation at ratio 1:100 (~6.4×10^7^ CFU) in mLB medium and kept at incubator 30°C using varied chromones concentrations in 96-well plates for 24 h in static conditions. A Multiskan EX microplate reader recorded cell OD at 620 nm for 24 h (Thermo Fisher Scientific, USA). The MICs of *V. harveyi s* and *V. parahaemolyticus* measured using broth method microdilution (Lee et al., 2022) with minor modifications. Briefly, both strains were diluted 1:100 (OD 0.1) in mLB overnight and treated with different concentrations (5, 10, 20, 50, 100, 200, 400, 500, and 1000 μg/mL) on 96-well plates. The MIC was determined as the treated concentration at which has no visible growth was perceived after 24 h of static incubation period at 30°C. The statistics represent the average of at least three independent cultures.

### 2.3 Evaluation of biofilm inhibition assay

The antibiofilm susceptibility of *V. parahaemolyticus* against chromone and its derivatives was assessed using crystal violet, which was modified slightly from the method described previously (Sathiyamoorthi et al., 2023). The overnight grown culture of *V. parahaemolyticus* and *V. harveyi* was prepared with mLB media at 1:100 dilution and then transferred to the 96-well plate with the total volume of 300 µL per well (SPL Life Sciences, Pocheon-si, Korea) and incubated for 24 h at 30°C under static circumstances in the presence of chromone and its derivatives (2, 5, 10, 20, and 50 µg/mL).Then three times splashed with sterile distilled water to exclude the non-adherent cells. Crystal violet at a concentration of 0.1% was introduced into individual wells to stain biofilm cells for 20 min. The attached crystal violet was dissolved in 95% ethanol after rewashing with distilled water. A Multiskan EX (Thermo Fisher Scientific) microplate reader were used to measure the quantity the biofilm cells at 570 nm. The mean of 6 duplicate wells was used to calculate the percentage of biofilm formation.

### 2.4 Dispersal assay

The biofilm dispersal assay were performed to disruptive cells with chromone and its derivatives of *V. parahaemolyticus* was evaluated. Briefly, after biofilm formation for 24 h at 30°C, planktonic cells were rinsed three times with phosphate-buffered saline (PBS, pH 7.4). Then, freshly prepared mLB media containing various concentrations (10, 20, 50, 100, 200, 400, and 500 µg/mL) were added to 96-well polystyrene plates containing *V. parahaemolyticus* biofilms and incubated at static conditions at 30°C for another 24 h. Then biofilm was dispersed by washing three times with water to get rid of the non-adherent cells and attained with crystal violet for 20 min, rinsing it with water followed by 95% ethanol. At OD_570_ nm, the absorbance was measured. The averages from 12 replicate wells were used to display the results.

### 2.5 Visualization of *V. Parahaemolyticus* in live imaging microscopy and scanning electron microscopy (SEM)

Two microscopic methods were used to perceive the impact of active compounds on biofilm formation by *V. parahaemolyticus*, as reported formerly (Kim et al., 2022b). *V. parahaemolyticus* was cultivated overnight and diluted with 1:100 in mLB medium for the live imaging. Subsequently, the mixture was distributed into six-well plates to facilitate attachment of biofilm formation. Then the plates were incubated at 30°C in the presence or absence chromone and its active derivatives 5, 10, and 20 µg/mL concentrations at static environments for 24 h. The used media was detached carefully from each well and rinsed three times with a PBS buffer solution. The iRiS Digital Cell Biofilms Imaging System (Logos BioSystems, Anyang, Korea) was utilized to analyze the biofilms at various magnifications. Utilizing the ImageJ imaging program (https://imagej.nih.gov/ij/index.html), the collected biofilm pictures were transformed into colored 3-D images.

SEM showed that the *V. parahaemolyticus* biofilms were formed on 0.5 × 0.5cm nitrocellulose membranes as described previously (Faleye et al., 2021) in the presence or absence 20 µg/mL of chromone derivatives under static condition for 24 h at 30°C. Consequently, cellulose membrane-adhering cells were fixed as follows: (i) they were fixed using (2.5%; glutaraldehyde) and (2%; formaldehyde) for an overnight period at 4°C; (ii) the samples were dehydrated with different ethanol dilutions (30, 50, 70, 80, 95, and 100%) for a 10 min interval; (iii) the critical point dryer was sputter coated along with platinum and gold; (iv) the specimens were examined at varying magnifications utilizing the (FESEM; S-4200 Hitachi Field Emission) scanning electron microscope (Hitachi Systems Ltd., Tokyo, Japan) operating at 10 kV (Sathiyamoorthi et al., 2023).

### 2.6 Swimming and swarming motility

For swimming motility, the bacterium *V. parahaemolyticus* was cultured on semi-solid mLB plates containing 0.3% of agar and chromone and its active derivatives were also added at concentrations of 5, 10, and 20 µg/mL. Then the overnight culture (1 µL) was placed in the middle of the agar plate and maintained at upright position. Therefore, for swarming motility, the mLB plates were supplemented with 0.5% agarose sealed and kept at an inverted position for 24 h at 30°C. Plates without the chromone derivatives was used as the control.

### 2.7 Yeast agglutination assay using *Saccharomyces cerevisiae*

The agglutination assay was studied using chromone derivatives as the earlier procedure (Sethupathy et al., 2020). *V. parahaemolyticus* overnight grown cultures (1:100) dilution were treated with chromone derivatives for 24 h at 30°C kept at 250 rpm. The cell pellets were adjusted to an approximate optical density of 0.5 OD, and 400 µL of the mixture was progressed to 14 mL tubes containing 1500 µL phosphate buffer solution and 500 µL concentration of 2% newly prepared *S. cerevisiae* (Sigma–Aldrich, St. Louis, USA). After gentle vortexing of the mixture for 5 s, the initial OD was measured at 600 nm using a UV-spectrophotometer (Optizen 2120UV, Korea). Subsequently, 25 min of incubation was kept at ambient temperature, 100 µL of the translucent supernatant was transferred into 96-well plates, and OD600 were measured. The following formula calculation showed agglutination as percentage: 100 × (1 − (OD_600before_/(OD_600after_)).

### 2.8 Hydrophobicity assay

The effects of chromone derivatives on the cell surface hydrophobicity of *V. parahaemolyticus* were explored using the ability of microbes to adhere to hydrocarbons as the earlier study reported (Sathiyamoorthi et al., 2021). The overnight grown culture was diluted (1:100). The diluted culture was treated with or without chromone derivatives at 5, 10, and 20 µg/mL and at that moment incubated at shaking 30°C at agitation 250 rpm for 24 h. After cultivation, 1 mL of the grown culture was r centrifuged at 12,000×*g* for 15 min. The pellets from bottom of the tubes were washed and redissolved in 4 mL of sterile PBS, and the bacterial cells OD 600 nm was adjusted to have ∼0.5 (denoted A_o_). Hexadecane (1 mL) was further added to the suspensions, vortexed robustly for 1 min, and incubated at room temperature to discrete the organic and aqueous phases. Subsequently, aqueous phase (1 mL) was removed carefully, and the final OD reading at 600 nm (Ai) was measured using optical UV/Vis-spectrophotometer (Optizen 2120UV, Korea). The resulting formula was used to accomplish the percentage of hydrophobicity: Hydrophobicity (H)% = (A_o_ − A_i_)/A_o_ × 100. The statistical values presented the mean standards of six distinct cultural groups.

### 2.9 Assay of protease production

Bacterial protease production was measured as previously reported (Sethupathy et al., 2020) with chromone derivatives compounds. *V. parahaemolyticus* was treated with chromone and its derivatives (5, 10, and 20 µg/mL) at 30°C at 250 rpm for 24 h. After 24 h incubation, the microtube was centrifuged at 13,000×*g* for 15 min and 75 µL of the aqueous phase were treated with (2% w/v; 125 µL azocasein) and incubated at 37°C for 30 min. Consequently, (trichloroacetic acid solution (10%); 600 µL) was added to the suspension in order to halt the proteolytic activity, and tubes were stored at −20°C for 30 min, and (700 µL; 1 M NaOH) was added to the suspension (Da et al., 2023). The absorbance of the mixture was measured at 440 nm. The average mean values from six independent cultures was measured.

### 2.10 Indole assay with different pH

Indole synthesis was tested in the presence chromone and its derivatives under different pH settings, as reported (Sathiyamoorthi et al., 2023). Briefly, the overnight grown culture was diluted with mLB (1:100) dilution and was treated with 5, 10, and 20 µg/mL with chromones for 24 h at 30°C and agitation 250 rpm. The media was initially adjusted to the neutral pH 7 and then it was changed to pH 5 (35% HCl) and pH 9 (5 N NaOH), respectively, before inoculation to determine the effects of pH on indole production and antibacterial activities. After 10 h incubation, cell culture (1 mL) was centrifuged at 11,000 ×*g* for 10 min. Subsequently, a volume of 1000 µL of the upper phase was mixed with 300 µL of Kovac’s solution (10 g; p-dimethyl amino benzaldehyde, 50 mL; hydrochloric acid, and 150 mL; amyl alcohol). The reaction was allowed to continue for approximately 2 min at room temperature. The top layer of 50 µL were carefully moved to a cuvette holding 1000 µL (HCl-amyl alcohol solution). The indole production was measured at 540 nm. The data represent the mean values from six independent cultures.

### 2.11 Analysis of the biological surfaces of squid and shrimp

The potential of chromone and its derivatives to prevent the *V. parahaemolyticus* and biofilm formation on surfaces of seafoods was investigated, as reported previously (Faleye et al., 2021). The squid was meticulously prepared by separating the main body and mantle detached from the hood and tentacles. In a sterile Petri dish, the body was sectioned into 1.5 × 1.5 × 0.5 cm pieces using a sterile scalpel. The squid fragments were cleaned using sodium hypochlorite, followed by multiple washes with sterile distilled water, and subsequently placed the fragments into a safety chamber cabinet for a duration of 1 h. They were then divided into groups and given the appropriate treatments: the squid surface was treated with *V. parahaemolyticus*; the other groups were treated with 20 µg/mL of the target compounds. All the samples were primarily infected at 5.6 ×10^5^ CFU/mL and left undisturbed for 24 h at 30°C at static condition. The samples were prepared for SEM examination as mentioned above.

Consistent with the earlier study, the cooked shrimp samples were used to evaluate the chromone derivatives to extend the prolong shelf life of the seafood (Sathiyamoorthi et al., 2023). Thawed shrimp samples (1.0 g) were cleaned using distilled water and then exposed to UV irradiation within a biosafety chamber cabinet (JSCB-1200SB; Korea) for approximately 30 min to kill any remaining background microorganisms (15 min on the front and back side). The shrimp were inoculated with *V. parahaemolyticus* by submerging them in a solution containing the bacterium for five min (6 log CFU/g). They were then allowed to dry for 10 min in the air of the biosafety chamber cabinet. The samples were divided after inoculation, then immersed for 15 min in chromone and its derivatives at 0, 5, 10, 20, or 50 μg/mL. After treatment, the shrimp were stored in aseptic bags at 4°C for a maximum of five days. In addition, shrimp were used in an experiment with mixed species for a period of 9 days using the same methodology. The samples were collected at regular intervals and vortexed violently to release the cells. The colony-forming units (CFU) was used to calculate the prevalence of *V. parahaemolyticus* and *V. harveyi* on shrimp.

### 2.12 *Caenorhabditis elegans* cytotoxicity assay

The toxicity assay employed the *C. elegans* strains *fem-1 (hc17)* and *fer-15 (b26)*, as reported previously (Lee et al., 2018). Already matured synchronized worms (approximately 30 worms) were transferred into individual wells of 96-well plates that contained buffer solution (M9). The worms were then subjected to varying altered concentrations of chromone and its derivatives, ranging from 0, 20, 50, 100, 200, or 400 μg/mL in the absence of *V. parahaemolyticus*. The plates were kept in an incubator for seven days at 25°C in static environment. The percentage of survival rate of the worms as determined by the nematode reactions to contact with the platinum wire, was used to express the toxicity of the derivatives. The experiment was kept for a week and monitored using an iRiS™ digital automated cell imaging system (Logos Biosystems in Korea). The experiment was conducted in triplicate, and mean values denoted the data.

### 2.13 To analyze seed germination with chromone derivatives

The effects of chromone and the two selected hit compounds on the germination of radish seeds (*Raphanus sativus*) seeds were investigated, as previously described (Ahmed et al., 2022). Briefly, the radish seeds were immersed in sterile distilled water and left to soak overnight, and then washed with distilled water and once ethanol (95%), and cleaned with a 3% sodium hypochlorite solution for 10 min. The seeds (n=0) were distributed evenly on each plate at different concentrations (0, 20, 50, 100, 200, or 400 μg/mL). The agar plates utilized in the experiment were Murashige and Skoog (0.86 g/L; MS) consisting of 0.7% agar. The plates were subjected to incubation at 25°C for five days in the presence of chromone and its derivatives. The seed germination images were recorded on the fifth day.

### 2.14 Quantitative (qRT-PCR) analysis

Gene expression studies were conducted on the chromone derivatives to gain vision into the potential mechanisms of action of these compounds as antibiofilm and antibacterial agents. In particular, a diluted overnight culture of *V. parahaemolyticus* (OD600 = 0.1; ~5.2×10^6^ CFU/mL) was cultured to OD600 = 1.0 in a 25 mL culture volume. Subsequently, the bacterial cells were inoculated with or without the active compounds at concentration of 20 μg/mL for 4 hr at 30°C with 250 rpm. RNase inhibitor was introduced to avoid RNA degradation during cell collection. Total RNA was purified using a Qiagen RNeasy minikit (Valencia, CA, USA).

Quantitative (qRT-PCR) was used to assess the level of gene expression involved in biofilm formation and virulence, such as *luxS aphA*, *opaR*, *tolC*, *tdh*, *fliA*, and, *vmrA* as described elsewhere (Faleye et al., 2021). *16S rRNA* was employed as a housekeeping gene without impacting its expression from chromone derivatives. StepOnePlus Real-Time PCR System and an SYBR Green qPCR Master Mix (Applied Biosystems, Foster City, USA) were used to implement the qRT-PCR (Applied Biosystems). The range of gene communication was standardized using the expression of 16S housekeeping genes. Four PCR cycles were performed with two independent cultures. The expression data for the reference and target genes were generated as threshold cycle (Ct) values. The final ddC_t_ were obtained by the comparing the target gene to the housekeeping in order to control variance among samples and the fold change was calculated as 2^(-ddC_t_).

### 2.15 Statistical evaluation

The experiments included three cultures and six duplicates. The data are mean values with ± SD. With a significance threshold of p < 0.05, the Student’s t-test were used to assess mean significance.

## 3 Results

### 3.1 Chromone derivatives inhibited biofilm formation of two *Vibrio* species

The antibacterial activities of 27 chromones against two *Vibrio* strains were initially determined using a minimum inhibitory concentration (MICs) assay. The trend in antimicrobial efficacy of the 27 chromones was similar between *V. harveyi* and *V. parahaemolyticus* (**Table 1**). In particular, the 6B3FC and 6C3FC had MIC of 20 µg/mL while 3-bromochromone, 3-bromo-6-chlorochromone, chromone-3-carbonitrile, 3-formyl-6-methylchromone, 3-formyl-6-isopropylchromone, 6-formyl-6-methoxychromone and 6-isopropylchromone-3-carbonitrile showed the MICs range from 50–200 µg/mL. On the other hand, the remaining 18 chromone derivatives did not show an inhibitory impact on planktonic cell growth even at >500 µg/mL (**Table 1**). In mixed culture of *Vibrio* species, the MIC of 6B3FC and 6C3FC was found to be 50 and 20 µg/mL, respectively.

**Figure 1:**
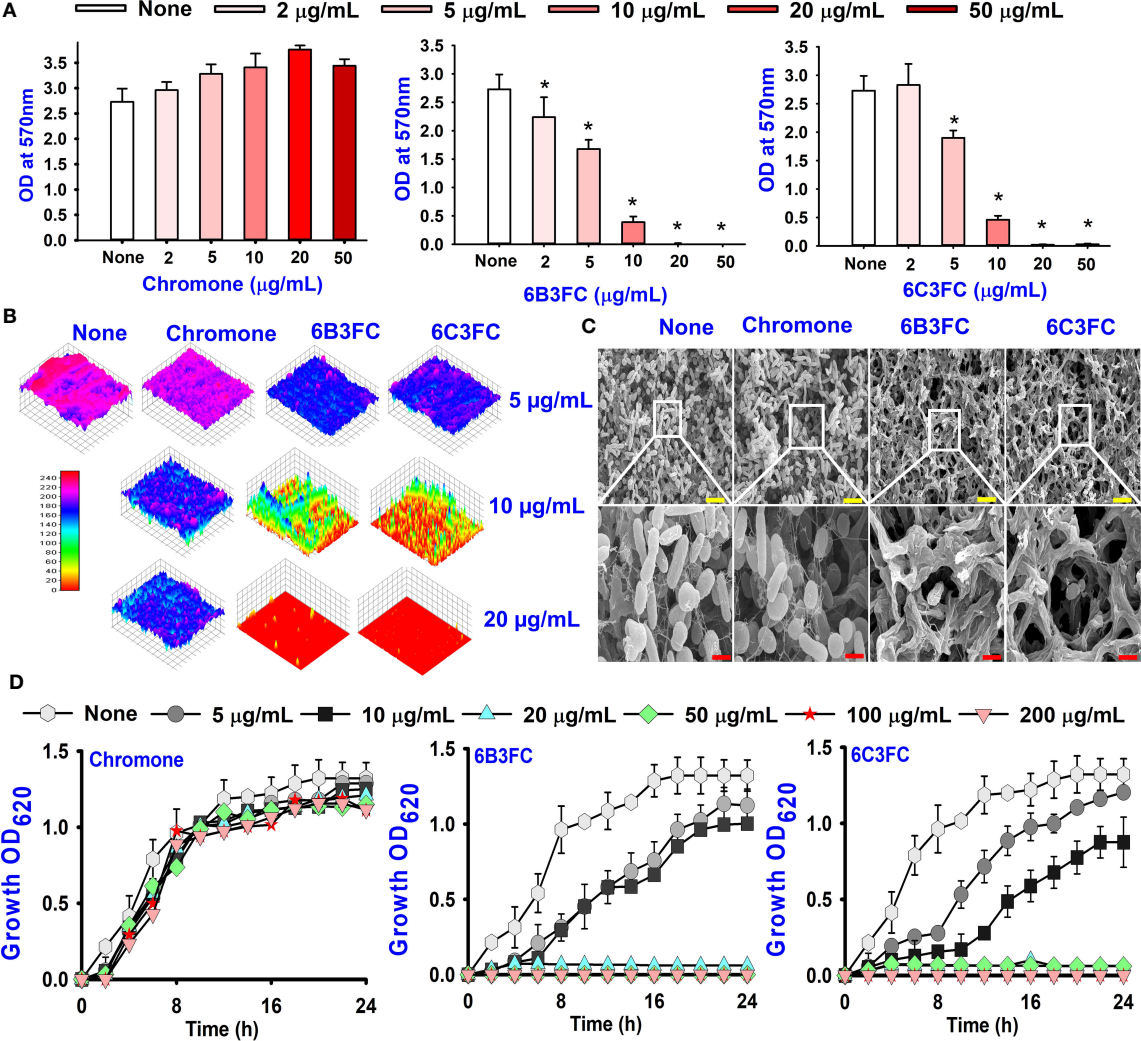
Biofilm inhibition of chromones derivatives against *V. parahaemolyticus* **(A)**. Microscopic visualization of *V. parahaemolyticus* biofilms inhibited by the selected chromones **(B)**. SEM images displayed the biofilm cells treated with chromone, 6B3FC, and 6C3FC **(C)**. The yellow and red bars indicate 6 and 1.5 µm, respectively. Planktonic cell growth curve of *V. parahaemolyticus* with chromone derivatives **(D)**. * indicates p < 0.05.

Also, the twenty-seven chromones were screened at 20 and 50 µg/mL concentrations to evaluate the antimicrobial potential of *V. harveyi* and *V. parahaemolyticus* (**Table 1**). Among the compounds tested, several halogenated chromones showed antibiofilm activities in both strains. For example, four hits, such as 6B3FC (**7**), 6C3FC (**13**), 3-formyl-6-isopropylchromone (**18**), and 3-formyl-6-methylchromone (**20**), at 50 μg/mL suppressed biofilm formation by the two *Vibrio* strains while the backbone of chromone had no antibiofilm activity (**Table 1**). Furthermore, the detailed study showed that chromone and its two most active chromone analogs (6B3FC and 6C3FC) dose-dependently decreased the biofilm formation in *V. parahaemolyticus* at 5, 10 and 20 µg/mL which matches with the antibacterial activities displayed (**Figure 1A**). In addition, our study evaluated the impact of two halogenated active compounds on a mixed culture of *Vibrio* species. The results showed that 6B3FC and 6C3FC slightly inhibited the mixed species biofilm at 2-10 µg/mL but was significant at ≥ 20 µg/mL suggesting that chromones derivatives possess broad-spectrum efficacy (**Supplementary Figure 3**). The antibiofilm effects of chromone derivatives were confirmed by live cell imaging and SEM. The cell imaging 3D mesh plot showed that 6B3FC and 6C3FC at 10 and 20 µg/mL reduced the thickness of biofilms significantly compared to the untreated (**Figure 1B**). SEM showed that the untreated control adhered to each other to form a biofilm largely covered by an extracellular mucous membrane substance and the bacteria with the usual rod-shaped of *V. parahaemolyticus* cells trapped in the matrix. On the other hand, the treated bacterial cells 6B3FC and 6C3FC at 20 µg/mL exhibited a clear reduction in the biofilm formation with fewer cells and extracellular material (**Figure 1C**). In addition, the two active chromone derivatives were monitored for cell growth curve of *V. parahaemolyticus* for 24 h. While chromone did not impede the cell growth, 6B3FC and 6C3FC at 5 and 10 µg/mL slightly decreased the cell growth with complete inhibition at 20 µg/mL (**Figure 1D**). Therefore, the 6B3FC and, 6C3FC were selected used for further experiments in this study.

The results of the dispersal assay demonstrated that chromone and its derivatives reduced the development of biofilm in a dose-dependent manner (**Supplementary Figure 2**). For instance, the development of biofilm was not prevented by chromone at a concentration of 100 µg/mL; however, the active compounds 6B3FC and 6C3FC suppressed the matured biofilms considerably at concentrations of at 50 & 100 µg/mL, respectively. Since the two hits compounds were investigated to focus on the ability to prevent their biofilm formation or eradicate established biofilm formation (**Supplementary Figure 2**).

### 3.2 Two formylchromones reduced the motilities, fimbriae, protease production, cell surface hydrophobicity, and indole production

**Figure 2:**
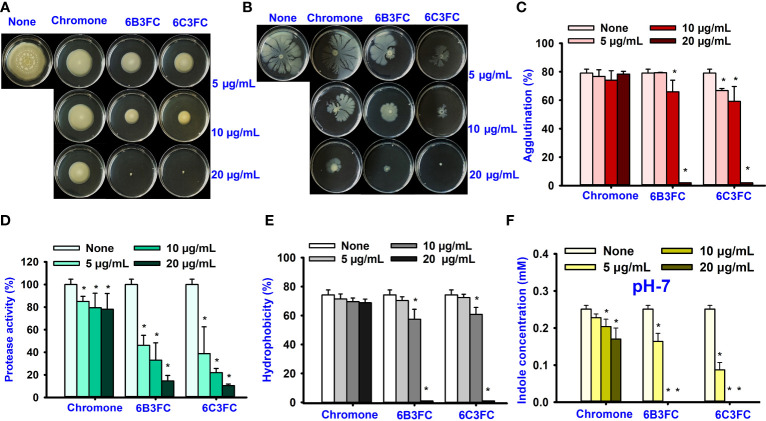
The effects of chromone derivatives on the surface motility of *V. parahaemolyticus*. Swim motility assay **(A)** and swarm motility assay **(B)**. Effects of chromone derivatives on fimbrial activity **(C)**, protease activity **(D)**, cell surface hydrophobicity **(E)**, and indole production treated pH 7 **(F)**. * indicates p < 0.05.

Motility is crucial in developing *Vibrio* infections to colonize host tissues by pathogenic bacteria (Silva Anisia et al., 2006). Hence, the ability of selected hits to suppress motility was investigated (**Figures 2A, B**). The swimming and swarming motility was reduced by chromone dose-dependently compared to untreated, whereas at 20 µg/mL 6B3FC and 6C3FC completely inhibited the motility. In particular, 6B3FC and 6C3FC at 10 µg/mL reduced the swimming motility by 33% and 30%, respectively, and the swarming motility by 62% and 46% (**Figures 2A, B**). The role of yeast agglutination was explored so that the effects of fimbria could be investigated. Two formylchromones (6B3FC and 6C3FC) decreased the fimbria activity at 10 µg/mL 13% and 20%, respectively, and showed complete inhibition at 20 µg/mL (**Figure 2C**).

The virulence of *Vibrio* spp. was positively influenced by extracellular proteases that had cytotoxic action against Chinese hamster ovary (CHO) and Vera cells (Lee et al., 2002). The selected hit 6B3FC and 6C3FC derivatives reduced the protease activity in a dose-manner manner, 85% to 89% at 20 µg/mL, while its backbone chromone did not affect (**Figure 2D**).

The cell surface charge affected bacterial adhesion to surfaces. *V. parahaemolyticus* cells that prefer negative-charged surfaces. Flagellar and outer surface membrane proteins also influenced *V. parahaemolyticus* in biofilm development. (Letchumanan et al., 2014; Mizan et al., 2016). As a result, the effects of chromone analogs on cell surface hydrophobicity were investigated. Two formylchromones (6B3FC and 6C3FC) at 10 µg/mL showed moderate inhibition, 17% and 13%, respectively, and would completely inhibit the hydrophobicity at 20 µg/mL (**Figure 2E**).

Indole is an important signaling molecule in various bacteria, including *V. parahaemolyticus* (Lee and Lee, 2010). This study evaluated the different pH 5, 7, and 9 levels to monitor indole production in *V. parahaemolyticus*. The indole level was much greater at pH 9 than at pH 5 and pH 7 (**Figure 2F** and **Supplementary Figure 1**). The findings suggested that pH is crucial to synthesize indole production because indole production is repressed by acidic pH, and indole production is favored by neutral and alkaline conditions. The two formylchromones (6B3FC and 6C3FC) reduced the indole production significantly in a dose-dependent manner.

### 3.3 Formylchromones inhibited biofilm formation on squid surface

**Figure 3:**
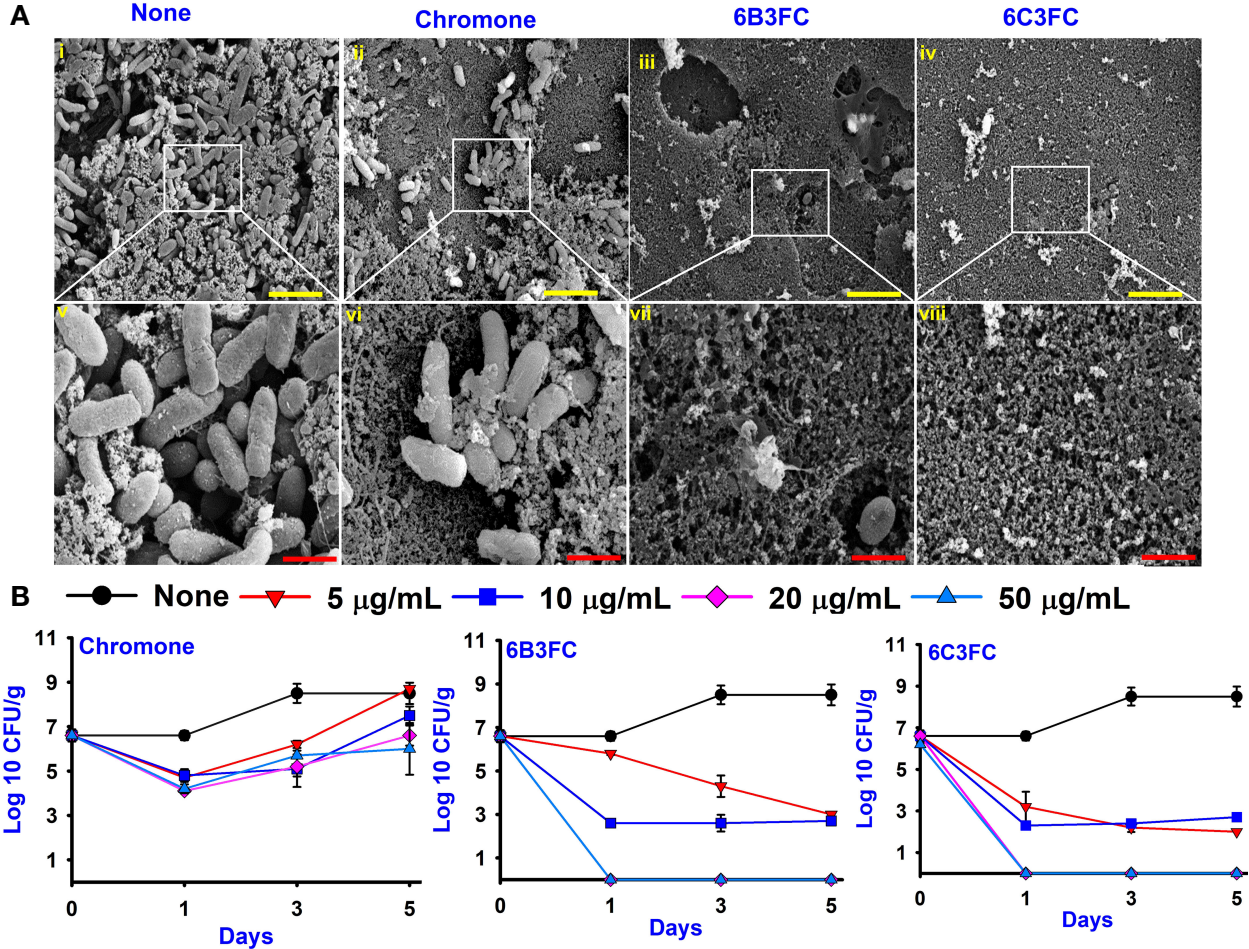
SEM images showed the antibacterial effect of chromone derivatives on the surfaces of squid (i-viii). None represents untreated *V. parahaemolyticus* (i, v) and active compounds treating with 20 µg/mL of chromone (ii, vi), 6B3FC (iii, vii) and 6C3FC (iv, viii) **(A)**. The yellow and red color scale bars represent 6 µm and 1.5 µm, respectively. The chromone derivatives showed the antimicrobial efficacy in a shrimp model **(B)**.

The ecological habitat of *V. parahaemolyticus* can be predominately associated with seafood. Therefore, the two active formlychromones could prevent biofilms formation by *V. parahaemolyticus* on the surfaces of squid. The untreated cells and chromone formed the clusters with mucous substances of *V. parahaemolyticus* cells (**Figures 3Ai,ii,v,vi**). While the treatment of two formlychromones at 20 µg/mL showed a reduced number of cells and prevented biofilm formation (**Figures 3Aiii,iv,vii,viii**).

In addition, the antibacterial and preservation efficacy of two formlychromones was investigated for five days using a cooked shrimp model. The untreated or chromone at 5–50 μg/mL could not affect CFUs of *V. parahaemolyticus*. However, 6B3FC and 6C3FC showed bactericidal effects at 20 and 50 μg/mL (**Figure 3B**). Specifically, at 20 and 50 μg/mL treatment with 6B3FC and 6C3FC, *V. parahaemolyticus* was not detected in the shrimp after one day. Additionally, we examined the preservative potentials of chromone derivatives in boiled shrimp model seeded with mixed species (*V. parahaemolyticus* and *V. harveyi*). Compared to the control, we observed that chromone at concentrations of 5-50 µg/mL could not restrict the growth of mixed species during 9 days of storage (8log_10_). However, 6B3FC and 6C3FC at 50/mL significantly reduced the mixed *Vibrio* species load by 4log_10_ within 1 day suggesting a bactericidal effect. Also, at 20 both compounds exhibited 2.5log_10_ reduction in the bacterial load (**Supplementary Figure 4**). These results suggests that the chromone derivatives could act as preservatives to extend the shelf life of seafood products.

### 3.4 Toxicity effect of chromones on plant seedling and *C. elegans* survival

**Figure 4:**
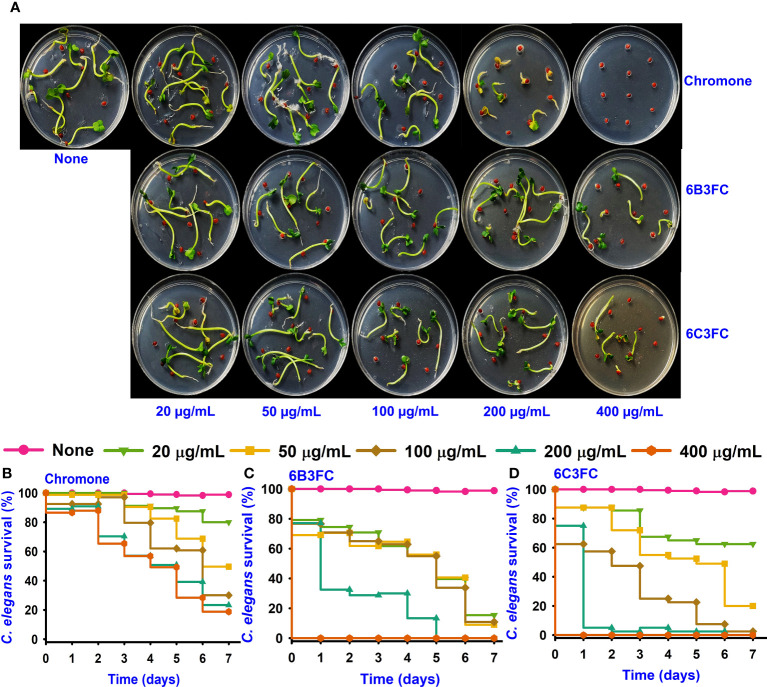
Toxicity of chromones on seedling **(A)**. Toxicity effects of chromone **(B)**, 6B3FC **(C)**, and 6C3FC **(D)** on *C. elegans*.

The seed germination assay was performed as a toxicity assessment for the environment. While chromone was not toxic at 20–100 µg/mL but showed some toxicity at 200–400 µg/mL, the formylchromones (6BCFC and 6C3FC) had no harmful effects on *R. sativus* seeding at any concentrations between 20–200 µg/mL, but they were mildly toxic at 400 µg/mL (**Figure 4A**).

The *Caenorhabditis elegans* model has been used extensively to screen the antimicrobial compounds. Different concentrations were given to healthy nematodes to examine the toxicity of chromone and two formylchromones. The results showed that chromone was less toxic than 6B3FC and 6C3FC in the nematode model. However, 6C3FC exhibited moderate toxicity at 50 µg/mL and 6B3FC were toxic to the nematodes 20-100 µg/mL (**Figures 4B–D**).

### 3.5 Formylchromones suppressed the genes of QS, biofilm, and virulence factor genes

**Figure 5:**
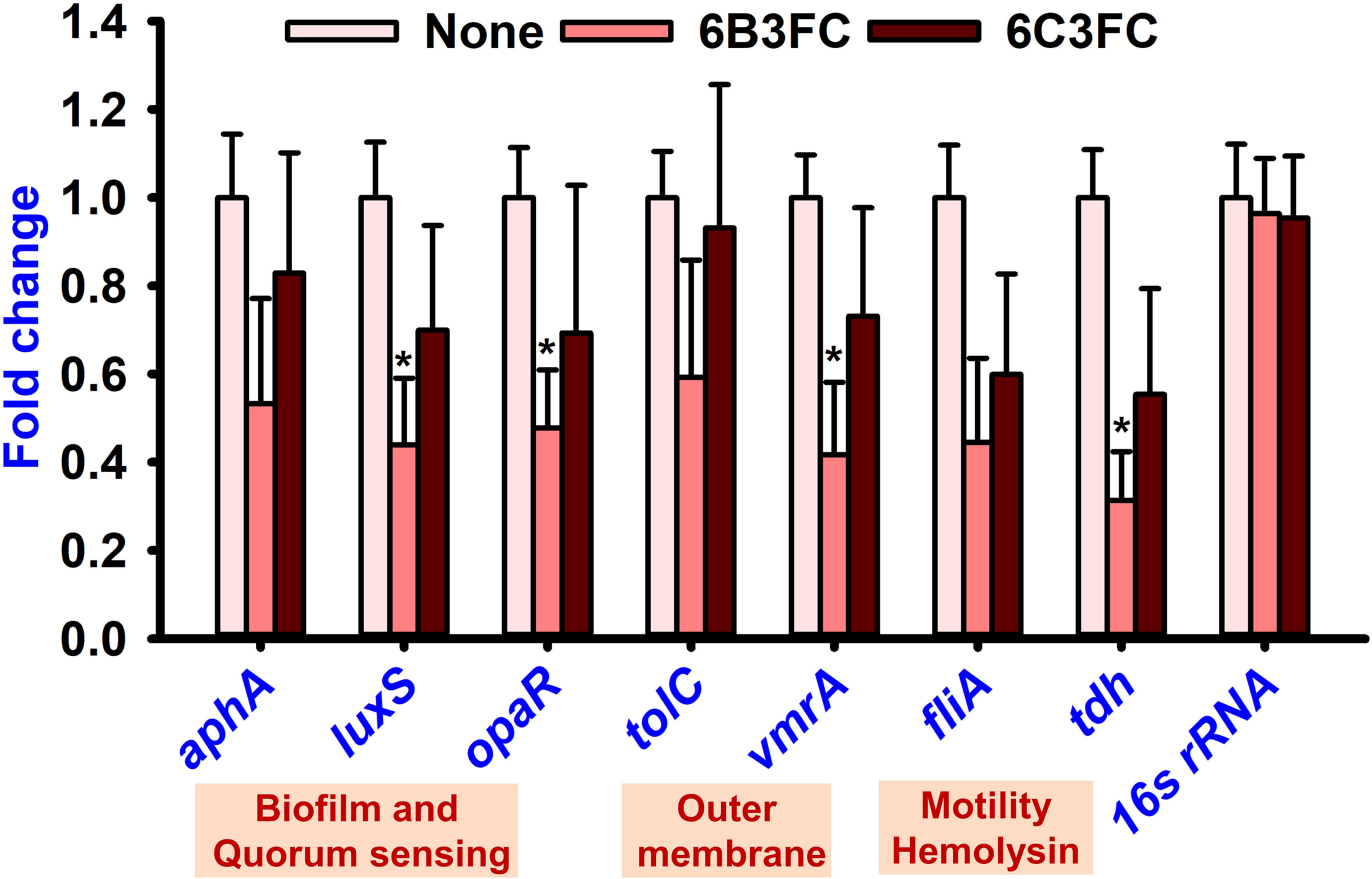
Gene expression of *V. parahaemolyticus* presence or absence of formylchromones. Compared to the non-treated controls, comparative expressions indicate that the transcriptional levels after treated with 6B3FC or 6C3FC at 20 μg/mL. * indicates p < 0.05.

qRT-PCR was performed with seven genes involved in biofilm formation, quorum sensing, membrane transport, pathogenicity, and secretion to understand the potential mechanisms of the antibiofilm and antibacterial activity. Only 6B3FC altered the expression of four genes, such as *luxS*, *opaR*, *vmrA*, and *tdh*, while 6C3FC marginally changed their expression (**Figure 5**). For example, 6B3FC reduced the expression of the genes (*luxS*, *opaR*, *vmrA*) by 0.6-fold and *tdh* by 0.7-fold changes.

## 4 Discussion

The halophilic bacteria known as *Vibrio* spp. are responsible for forming biofilms on the surface of many types of seafood. The bacteria are often found in coastal areas and the marine environment. In particular, the bacteria inside the biofilms have 10 to 1000 times more resistant than bacterial planktonic cells (Su and Liu, 2007; Lee et al., 2019). Recently, natural compounds have attracted increasing attention as antimicrobial agents to prevent biofilm formation. They are appealing sources of phytochemicals to fight against bacterial infections, including *V. parahaemolyticus* (Nabavi et al., 2015).

Chromone, also known as 1, 4-benopyrone has a keto association replaced on the pyran ring. It has many biological activities (Duan et al., 2019; Semwal et al., 2020). Chromone carboxamide derivatives, which are members of this large family, might exhibit intriguing applications, such as the inhibition of the breast cancer protein (Roussel et al., 2020), Alzheimer’s (Suwanhom et al., 2020), and calpain (Kim et al., 2011), in addition to antioxidant properties. Furthermore, 3-formylchromone is a significant precursor used in synthesizing chromone derivatives and is easily prepared via the Vilsmeir–Haack reaction (Nohara et al., 1973; Mohsin et al., 2020). The effects of the 3-formyl group substituted at the C-6 position of chromones appear to have cytotoxicity against tumor-specific cell lines (Kawase et al., 2007). In addition, the chromone derivative CM3a inhibited biofilm formation and showed the antibacterial activity in *Staphylococcus aureus* (Zhan et al., 2021).

In this present study, various chromone derivatives revealed differential antimicrobial activity against *V. parahaemolyticus* and *V. harveyi* (**Table 1**). Notably, two halogenated formylchromones (6B3FC and 6C3FC) showed antibacterial activity, but chromone could not inhibit their biofilm formation (**Figure 1A**). The chromone had a MIC of >500 µg/mL, and its derivatives 6B3FC and 6C3FC had a low MIC of 20 µg/mL (**Table 1**). Their low MIC of two active formylchromone was the main reason for biofilm inhibition. The observed MIC for 6B3FC and 6C3FC was better than the antimicrobial activity of 5,7-dihydroxychromone-3α-d-*C*-glucoside (125−1000 μg/mL) against *Pseudomonas aeruginosa*, *Staphylococcus aureus*, *Escherichia coli*, and *Salmonella enterica* (Do Nascimento et al., 2020). Similarly, it also showed better potency than 4-chloroindole and 4-nitrocinnamaldehyde (MIC 50 µg/mL) against *V. parahaemolyticus* (Faleye et al., 2021; Sathiyamoorthi et al., 2021). Our result does show that the biofilm inhibition observed for the 6B3FC and 6C3FC were due to their antibacterial effects against the bacterium. Furthermore, the optical microscopy and SEM images confirmed the decline in the aggregate of bacterial cells and inhibition of biofilms formation of *V. parahaemolyticus* by 6B3FC and 6C3FC (**Figures 1B, C**).

Pathogenic bacteria use pili or fimbriae to invade host tissues (Mccarter, 1999). The present study found that the two formyl chromone derivatives, 6B3FC and 6C3FC, reduced the swimming and swarming motility (**Figures 2A,B**). Flagella, pili, non-fimbrial surface proteins, and surface polysaccharides influence the adhesion of *Vibrios* to surfaces in its native environment and during opportunistic infections. Oyster *Vibrio* spp. type IV pili assist biofilms to grow on biotic and abiotic surfaces, according to various Gram-negative pathogens (Paranjpye Rohinee et al., 2007; Karan et al., 2021). *V. parahaemolyticus* has a polar and lateral flagellar system to facilitate efficient free-floating planktonic bacterial cells with strong biofilm formation in swimming and swarming motility (Takekawa et al., 2020). The sigma factors and transcription regulators control the expression of flagellar genes and sodium proton motive forces, respectively. Polar flagella continue to be expressed in liquid media. In contrast, the lateral flagellar genes were stimulated by solid surface motility. They encountered nutrient iron deficiency in outer membrane protein, the presence of calcium, and suppression of polar flagellum (Gode-Potratz Cindy et al., 2010). In this study, two active formylchromones, 6B3FC and 6C3FC, decreased the swimming and swarming motility.

The two formylchromones prevented the foodborne infections from adhering to bacterial surfaces with decreasing growth of biofilm formation. In addition, at 20 μg/mL, both 6B3FC and 6C3FC prevented the fimbrial activity and hydrophobicity that were essential for the growth of bacterial biofilms (**Figures 2C, E**). Based on these findings, two formylchromones potentially limit fimbrial formation, which is crucial to biofilm inhibition. Furthermore, the hydrophobicity index also varies under environmental stress and dietary deficits and is a critical virulence factor for intercellular communication (Barrasso et al., 2022). The ability of *V. parahaemolyticus* to produce biofilms correlate favorably with the hydrophobicity of its cell surface, suggesting that these two formylchromones play a role in this phenomenon. The pathogenic *Vibrios* spp. produced many extracellular proteases involved in the virulence factor of host proteins (Khouadja et al., 2013). Because 6B3FC and 6C3FC inhibited the protease production (**Figure 2D**), they could diminish the pathogenesis of *Vibrio* infection.

Indole is a metabolite of tryptophan, which is a responsible quorum-sensing molecules produced by bacteria, such as *V. parahaemolyticus* and *Escherichia coli* (Lee and Lee, 2010). And, an increase in Na^+^ in an decarboxylase of oxaloacetate transmuted the *V. cholerae* might be the underlying the mechanism for the indole showed negativity against virulence decrease in the *V. cholerae* mutant (Snell, 1975). Furthermore, indole production was influenced by environmental conditions and including temperature and pH. Indole synthesis was inhibited at low pH and was more favorable for alkaline pH (Lee et al., 2007; Hu et al., 2010). Similarly, the findings demonstrated increased indole synthesis at pH 9 (**Figure 2F**). On the surface of seafood, particularly squid and shrimp, *V. parahaemolyticus* can suppress biofilm formation (**Figures 3A, B**). After treatment with 6B3FC and 6C3FC, no biofilm cells were visible on the seafood surface, indicating that they effectively prevented biofilm development by pathogenic bacteria. The toxicity of chromone and its two active formylchromones 6B3FC and 6C3FC in the seed germination models of *Raphanus sativus* and *C. elegans* (**Figures 4A, B**). According to previous observations that are comparable, 5,7- dihydroxychromone prevents the germination of *Abutilon theophrasti* Medic seeds (Spencer and Tjarks, 1985; Tăbăcaru et al., 2019). This *C. elegans* and seed germination methods used in this study are suggestive and we recommend a more detailed toxicity examination in more advanced models such as zebra fish.

The formylchromone 6B3FC suppressed the expression of *opaR*, *luxS*, *vmrA*, and *tdh* (**Figure 5**). In the bacterium, *V. parahaemolyticus*, related to quorum sensing genes (*opaR* and *luxS)* are crucial regulators of bacterial biofilm formation (Han et al., 2022). Two key regulatory elements, *opaR* and *aphA*, are responsible for maintaining the integrity of the *luxS* QS system. For example, this system regulates genes for virulence factors involved in biofilm formation bacterial colonization (*luxS*, *opaR*), thermostable direct hemolysis (*tdh*), and outer membrane (*vmrA*) (Chen et al., 2020; Faleye et al., 2021; Hu et al., 2022). Therefore, the formylchromone can suppress the virulence factor and persistence of *V. parahaemolyticus* in squid and shrimp.

## 5 Conclusions

This study discovered two chromone derivatives namely 6B3FC and 6C3FC as potent antibacterial and antibiofilm agents against *V. parahaemolyticus* and *V. harveyi*. In addition, they inhibited the swimming motility, protease activity, fimbrial agglutination, hydrophobicity, and indole production at 20 µg/mL which impaired the growth of the bacteria. They also inactivated the proliferation of *V. parahaemolyticus* in shrimp and squid models. Interestingly, they prevented the mixed biofilm interaction involving *V. parahaemolyticus* and *V. harveyi*. These findings were validated by microscopic observations, phenotypic changes, transcriptomic and toxicity analysis. Overall, chromone derivatives could be used as an alternative method to regulate the biofilm of *Vibrio* species in food systems and risk of foodborne infections caused by these pathogens.

## Data availability statement

The original contributions presented in the study are included in the article/**Supplementary Material**. Further inquiries can be directed to the corresponding author.

## Author contributions

ES: Investigation, Methodology, Formal analysis, Writing—original draft. JL and J-HL: Conceptualization, Writing—review & editing, Project administration, Funding acquisition and Resources. All authors contributed to the article and approved the submitted version.
