# Supplementary material for: Antimicrobial and antibiofilm activities of formylchromones against Vibrio parahaemolyticus and Vibrio harveyi
Source: Front Cell Infect Microbiol. 2023 Aug 17;13:1234668. doi: 10.3389/fcimb.2023.1234668 (PMC10471482; doi:10.3389/fcimb.2023.1234668)
Supplement: Supplementary file 1 [file DataSheet_1.docx]

**Supplementary Material**

**Antimicrobial and antibiofilm activities of formylchromones against *Vibrio* *parahaemolyticus* and *Vibrio harveyi***

Ezhaveni Sathiyamoorthi^a^, Jin-Hyung Lee^a^, Yulong Tan^b^, and Jintae Lee^a,^*

^a^School of Chemical Engineering, Yeungnam University, 280 Daehak-Ro, Gyeongsan, 38541,

Republic of Korea

^b^Special Food Research Institute, Qingdao Agricultural University, Qingdao, China

*Corresponding Author

E-mail: jtlee@ynu.ac.kr. Tel.: +82-53-810-2533. Fax: +82-53-810-4631


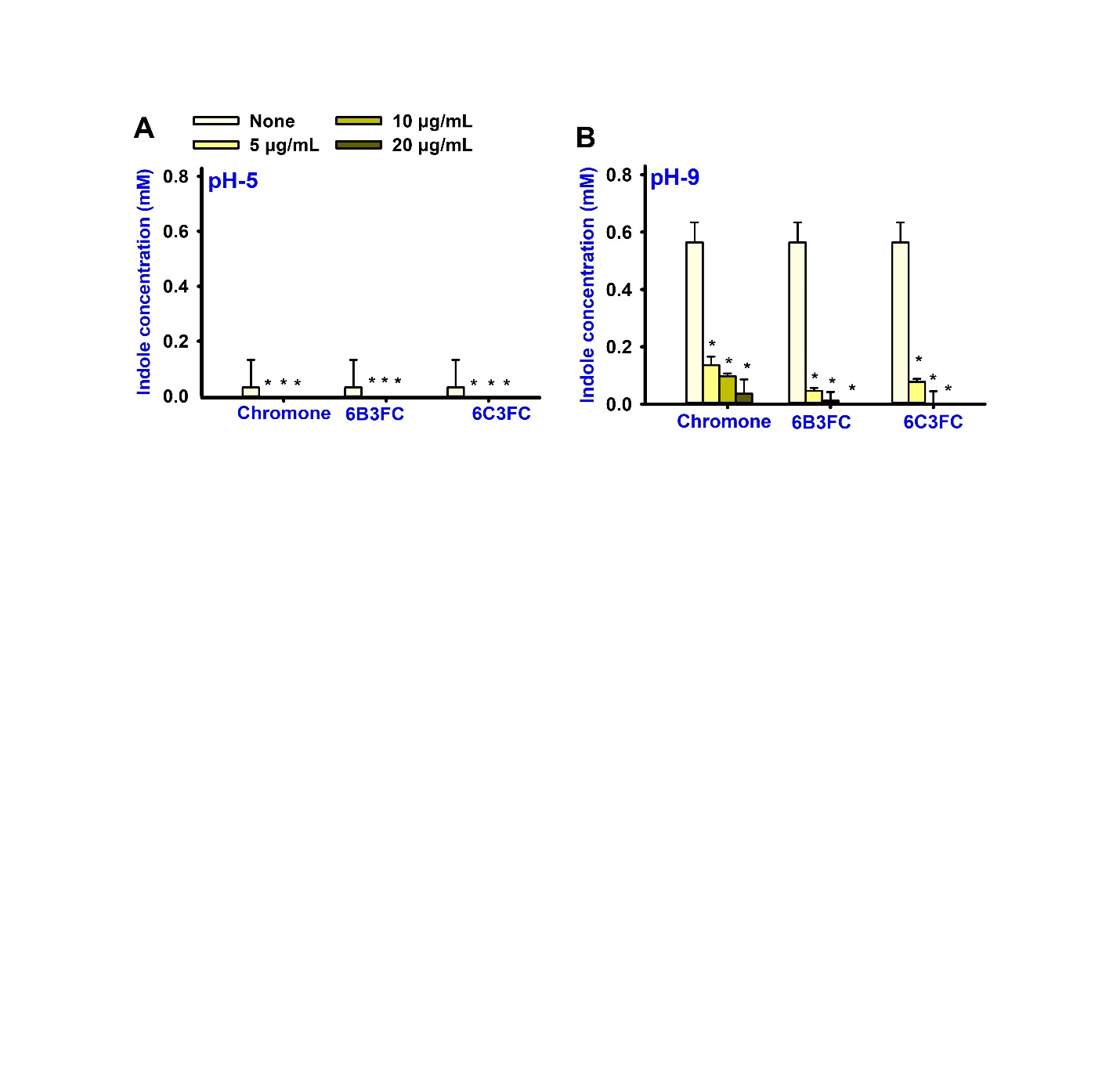


**Supplementary Figure 1:** Indole production treated with different chromone derivatives at pH 5 (A) and pH 9 (B). The error bars and asterisks (*) represent the standard deviation and significant differences (*p* <0.05), respectively, vs. the non-treated controls.


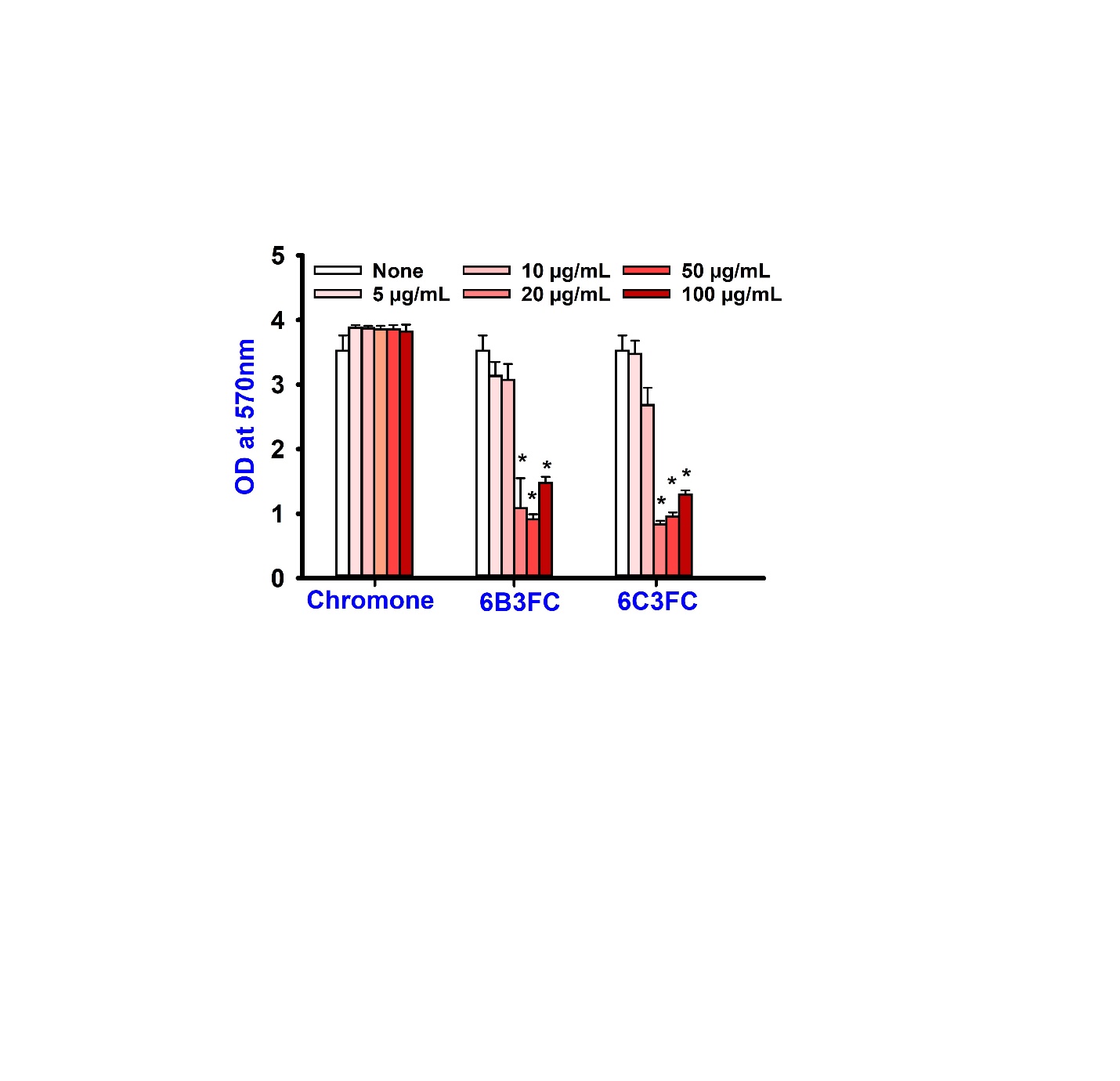


**Supplementary Figure 2:** Effect of dispersal assay on selected chromone and its derivatives compounds.


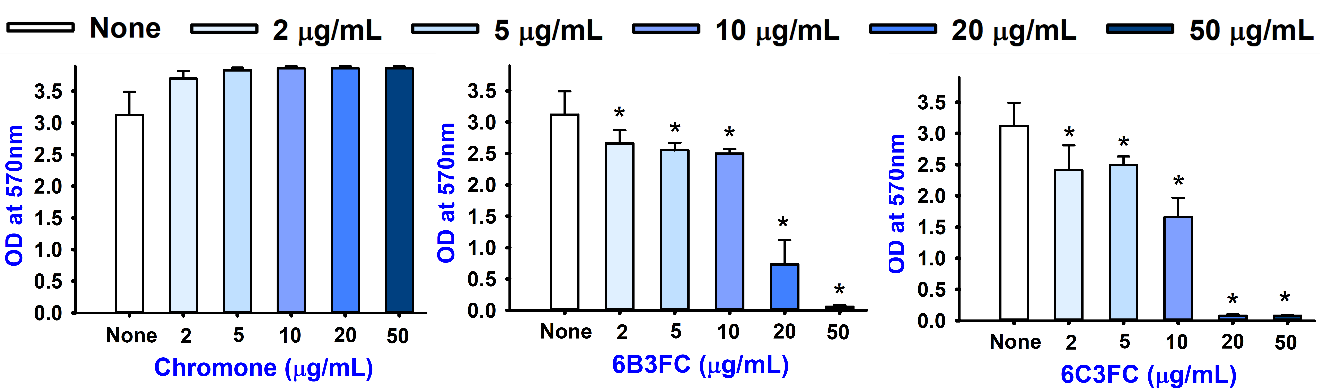


**Supplementary Figure 3**: Effects of chromone and its active derivatives on biofilm formation against mixed *V.parahaemolyticus* and *V. harveyi* species.


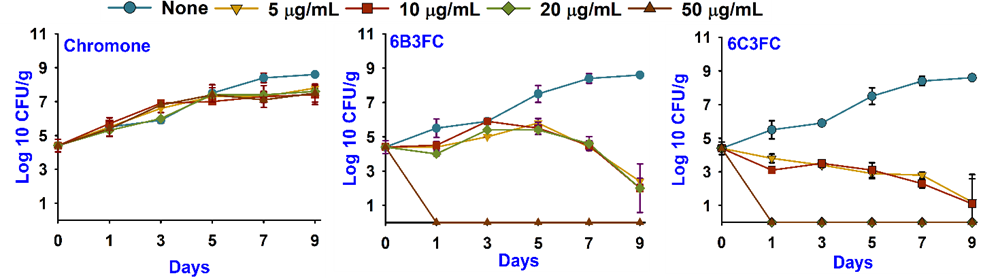


**Supplementary Figure 4**: The antibacterial efficacy of chromone derivatives in a shrimp model during storage of mixed *Vibrio* species.
